# Evaluating the accuracy of survey data: a case study of COVID-19 vaccination rates in Germany

**DOI:** 10.1186/s12874-025-02702-2

**Published:** 2025-10-22

**Authors:** Karolina von Glasenapp

**Affiliations:** GESIS – Leibniz Institute for the Social Sciences, Mannheim, Germany

**Keywords:** Survey design, Data quality, Accuracy, Adjustment weights, COVID-19 vaccination rate

## Abstract

**Background:**

Surveys are an important source of timely and comprehensive population health data and play a crucial role in public health research and policymaking, as shown during the COVID-19 pandemic. However, the reliability of survey data depends on their accuracy, which is often difficult to assess due to the limited availability of benchmark data. This study evaluates the accuracy of survey estimates of the COVID-19 vaccination rate in Germany and examines the impact of survey design and adjustment weights on accuracy.

**Methods:**

I compared survey estimates of the COVID-19 vaccination rate from multiple surveys conducted in Germany between 2021 and 2022 against administrative data on the vaccination rate as an external benchmark. Accuracy was assessed by calculating absolute and relative deviations between survey estimates and the administrative data. Further, I analyzed accuracy differences based on survey design, focusing on sampling procedure and survey mode, and I examined whether adjustment weights improved estimation accuracy.

**Results:**

The accuracy of survey estimates varied over time, with early surveys underestimating the vaccination rate and later surveys tending to overestimate it. Probability-based mixed-mode or personal interview surveys yielded more accurate estimates than did other survey designs. While some nonprobability web surveys performed well, their accuracy varied considerably. The application of adjustment weights generally improved estimation accuracy, suggesting that this weighting technique effectively addressed some sources of survey error.

**Conclusions:**

Survey-based estimates of COVID-19 vaccination rates should be interpreted with caution due to widespread inaccuracies, particularly overestimation. Probability mixed-mode and personal interview surveys produced more accurate estimates, underscoring the importance of robust survey methodologies. The fact that I found that adjustment weights enhanced accuracy highlights their value in survey research. These findings provide valuable insights for public health researchers and survey methodologists in designing and interpreting health-related survey data.

**Supplementary Information:**

The online version contains supplementary material available at 10.1186/s12874-025-02702-2.

## Background

Surveys have proved to be an important source of population information on health status, health determinants, and access to healthcare. The COVID-19 pandemic underscored their critical role, as surveys enabled researchers to monitor the rapidly evolving health situation and supported policy makers in combating the crisis. Examples of such studies include the COVID-19 Household Pulse Survey in the United States [1], the Household Impacts of COVID-19 Survey in Australia [2], the Coronavirus Infection Survey in the United Kingdom [3], and the COVID-19 Snapshot Monitoring in Germany [4].

The accuracy of survey data is a prerequisite for reliable and unbiased findings, but its empirical evaluation is challenged by the lack of benchmark data for comparison. Accuracy assessments focus mostly on comparing distributions of sociodemographic variables in the samples with external benchmarks provided by official statistics [5–7] and on comparing self-reported voting behavior with the actual election outcome as a benchmark [8–10]. Together, these studies show that accuracy varies largely across surveys.

According to the well-established total survey error (TSE) framework, accuracy of survey data can be improved through different actions at each stage of the survey process [11–13]. At the design stage, the choice of two key survey design characteristics – sampling procedure and survey mode – has a decisive impact on survey accuracy. Regarding sampling procedure, multiple studies have concluded that probability surveys are more accurate than nonprobability surveys [6–9, 14, 15], a result reached also in a meta-analysis by Cornesse and Bosnjak [16]. Regarding survey mode, the evaluation of its impact on accuracy is complex because it influences various types of survey error. Mode-specific errors include coverage error, such as the exclusion of certain population groups (e.g., the offline population in web-based surveys [17–19]), and measurement error, such as social desirability bias in sensitive questions (particularly in interviewer-administered surveys [20–22]. Despite the extensive literature examining individual error sources, only few studies have evaluated the overall impact of survey mode on survey accuracy [23, 24].

Moving to the stage of post-survey adjustments, survey weights can potentially improve survey accuracy. However, findings on the impact of adjustment weights on accuracy are mixed. Whereas some studies have found that adjustment weights improved accuracy [25–28], others have observed little difference in or a deterioration of accuracy [5, 6, 10, 14]. Furthermore, some studies have shown that the impact of adjustment weights differs across surveys, outcome variables, and the methods of comparison used for the assessment of accuracy [7, 15, 29, 30].

The provision of accurate information is a general requirement for public health studies, but the COVID-19 pandemic introduced additional challenges for survey research. First, the goal of maximizing accuracy was constrained by the additional requirement for timely delivery of results. As multiple studies have indicated, these parallel requirements pose the danger of trading established survey procedures for those with lower accuracy but higher speed [31–34]. In addition, contact restrictions limited the feasibility of the traditional interviewer-administered surveys and supported the use of self-administered survey modes. Assessing the accuracy of studies conducted during the pandemic — and the link between survey design and accuracy— is therefore of particular importance. For this purpose, I use data on a health-related substantive measure of high policy relevance, the COVID-19 vaccination rate. Focusing on surveys conducted between 2021 and 2022 in Germany, I investigate the following research questions:

- RQ1. How accurate were the survey estimates of the COVID-19 vaccination rate in Germany?
- RQ2. Did the accuracy of the survey estimates of the COVID-19 vaccination rate differ by survey design?
- RQ3. Did the application of adjustment weights improve the accuracy of the survey estimates of the COVID-19 vaccination rate?

By addressing these questions, I seek to provide a comprehensive evaluation of survey accuracy under the exceptional conditions of the COVID-19 pandemic and thereby offering valuable insights for both public health researchers and survey methodologists. In the following, I first provide contextual information on the COVID-19 vaccination campaign in Germany. Next, I introduce the data sources, measures, and methods used in this study. I then present the empirical results, discuss them in relation to existing research, and acknowledge the study’s limitations. I conclude with a summary and offer recommendations derived from the study.

## COVID-19 vaccination campaign in Germany

**Fig. 1:**
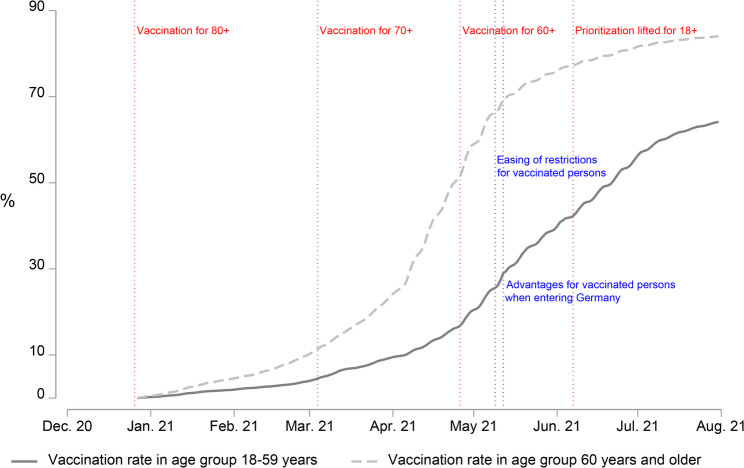
Timeline of the COVID-19 vaccination campaign in Germany: Vaccination rate (at least 1 dose) and selected policies Data sources: Vaccination rates – Robert Koch Institute (RKI) [38] and German Federal Statistical Office (Destatis) [39]; vaccine availability and policies – German Federal Ministry of Health chronicle [36] and the Oxford COVID-19 Government Response Tracker [40]

In Germany, COVID-19 vaccinations played an important role in the fight against the spread of the virus. Figure 1 maps some of the important milestones in the vaccination campaign in Germany between December 2020 and August 2021. The COVID-19 vaccination campaign began in Germany in late December 2020. The first vaccines were administered to the priority groups, including the elderly, those with high-risk health conditions, residents and staff of long-term care facilities, and selected occupational groups with increased exposure risk [35]. Eligibility was defined not only by residence status but also included individuals insured under the German health system or employed in Germany. Over the following months, the vaccines became available for younger age groups in the general population, as the events marked in red in Fig. 1 show. In June 2021, the prioritization order was lifted. From this point forward, all adults aged 18 or over living, working, or insured in Germany, regardless of citizenship, became eligible for COVID-19 vaccination [36, 37].

The development of the vaccination rate (gray lines in Fig. 1) reflects the accessibility of the vaccine. For both depicted age groups, the proportion of those vaccinated at least once grew until August 2021. During this time, the importance of vaccination to mitigate the spread of the virus was continuously emphasized by policymakers and encouraged by several political actions. Two examples of such actions introduced in May 2021 – the easing of restrictions for vaccinated persons and the introduction of advantages for vaccinated persons when entering Germany – are marked in blue in Fig. 1. Further policies and vaccination efforts followed in autumn and winter of that year, as the pace of the increase in the vaccination rate started to slow down and another pandemic wave arose [36, 41].

## Data and methods

### Data

The analysis is based on two data types – survey data and official health statistics. For the identification of relevant surveys, I relied on the SDCCP 1 dataset, a systematic collection of academic quantitative surveys conducted in Germany between March 2020 and December 2021 [42, 43]. This dataset covers a total of 717 surveys clustered within 183 survey programs. For the present study, I systematically screened the dataset for surveys that included a question on the respondent’s COVID-19 vaccination status. Furthermore, I included only surveys that covered the general resident population in Germany (based on the defined target population), excluding those aimed at narrower subgroups. The search within the SDCCP 1 dataset and the subsequent request for datasets resulted in 50 eligible surveys with available data clustered within 18 survey programs (e.g., rounds or waves).

To acknowledge the considerable differences in vaccine availability – and thus in the vaccination rate – by age, I divided the analysis into two age groups – 18–59 years and 60 years and older. The survey vaccination rate was calculated for each age group. However, some surveys did not allow a division into these two age groups, as either the age span of the survey target population differed from the required span, or information on respondents’ exact ages was not available. Thus, the final analytical samples for RQ1 and RQ2 comprised 34 surveys for the age group 18–59 years and 50 surveys for the age group 60 years and older. For the analyses for RQ3, I included only probability and non-probability surveys that provided adjustment weights in the datasets, allowing for a comparison of estimates with and without adjustment weights. Consequently, the analytical datasets for RQ3 were further reduced to 23 surveys in the age group 18–59 years and 39 surveys in the age group 60 years and older.

The official health statistics that served as benchmark data for my study were obtained from the Robert Koch Institute (RKI), a German public health institute that collected administrative data during the COVID-19 pandemic. Specifically, the benchmark data employed in the analyses included the number of vaccines administered daily [38]. To calculate the benchmark vaccination rate, I combined the benchmark data with population figures provided by the German Federal Statistical Office (Destatis) [39].

The analyses presented in this study are based on a customized dataset specifically created for this purpose. For each included survey, the dataset includes the survey estimate of the vaccination rate, variables capturing survey design features (as provided in the SDCCP 1 dataset), and the corresponding merged benchmark vaccination rate.

### Measures and methods

The measure of interest in my analyses is the COVID-19 vaccination rate defined as the percentage of the population that had received at least one dose of the vaccine. In the surveys, the vaccination rate represents the percentage of respondents who reported receiving at least one dose of the vaccine and refers to the respective fieldwork periods. In the benchmark data, I calculated the vaccination rate by dividing the number of first vaccine doses administered by the population number. As the benchmark vaccination rate was available on a daily level, I decided on a match based on the mean benchmark vaccination rate over the respective fieldwork period. Comparing both values, I calculated the absolute accuracy as the percentage point difference in the vaccination rate between the survey estimate and the benchmark value. With this definition, positive values refer to the overestimation and negative values to the underestimation of the vaccination rate by surveys. I note that official health statistics provide the best available benchmark for vaccination rates but may still contain errors. They should therefore not be regarded as perfectly accurate representations of the population parameter. This issue is further addressed in the Discussion.

In addition, I conducted two robustness checks (RCs) to test the sensitivity of the absolute accuracy measure matched with the mean benchmark vaccination rate. First, instead of the mean benchmark vaccination rate over the fieldwork period, I used the benchmark vaccination rate on the first day of fieldwork (RC1). My rationale for this approach was that the proportion of survey interviews completed at the beginning of the fieldwork period might have been high compared with the days and weeks that followed [44, 45]. Second, instead of absolute accuracy, I calculated relative accuracy in percent, defined as the difference between the survey estimate and the benchmark divided by the survey estimate (RC2). With this measure, I aimed to take into account the difference in vaccination rate levels over time. For instance, a small absolute inaccuracy of a few percentage points at the beginning of the vaccination campaign might have translated into a comparatively high relative inaccuracy in percent if the overall – then low – levels of the vaccination rate had been considered.

For the analysis of the association between accuracy and survey design (RQ2), I evaluated two key survey design features – sampling procedure and survey mode. The combinations of the two features in my sample yielded six survey design groups: (1) probability surveys conducted via computer-assisted personal interviewing (CAPI); (2) probability surveys conducted via computer-assisted telephone interviewing (CATI); (3) probability surveys conducted via computer-assisted web interviewing (CAWI); (4) probability mixed-mode surveys conducted via paper-and-pencil interviewing (PAPI) and CAWI; (5) probability mixed-mode surveys conducted via CATI and CAWI; and (6) nonprobability surveys conducted via CAWI. Due to the low number of cases, I combined both types of probability mixed-mode surveys into a single group for the interpretation of the results.

For the analysis of the association between accuracy and survey weights (RQ3), I compared the accuracy of survey estimates with and without adjustment weights. In both cases, design weights were applied when available for probability surveys. For the analyses addressing RQ1 and RQ2, I applied all weights provided in the datasets (design and adjustment).

## Results

### RQ1 – Overall accuracy of survey estimates

**Fig. 2:**
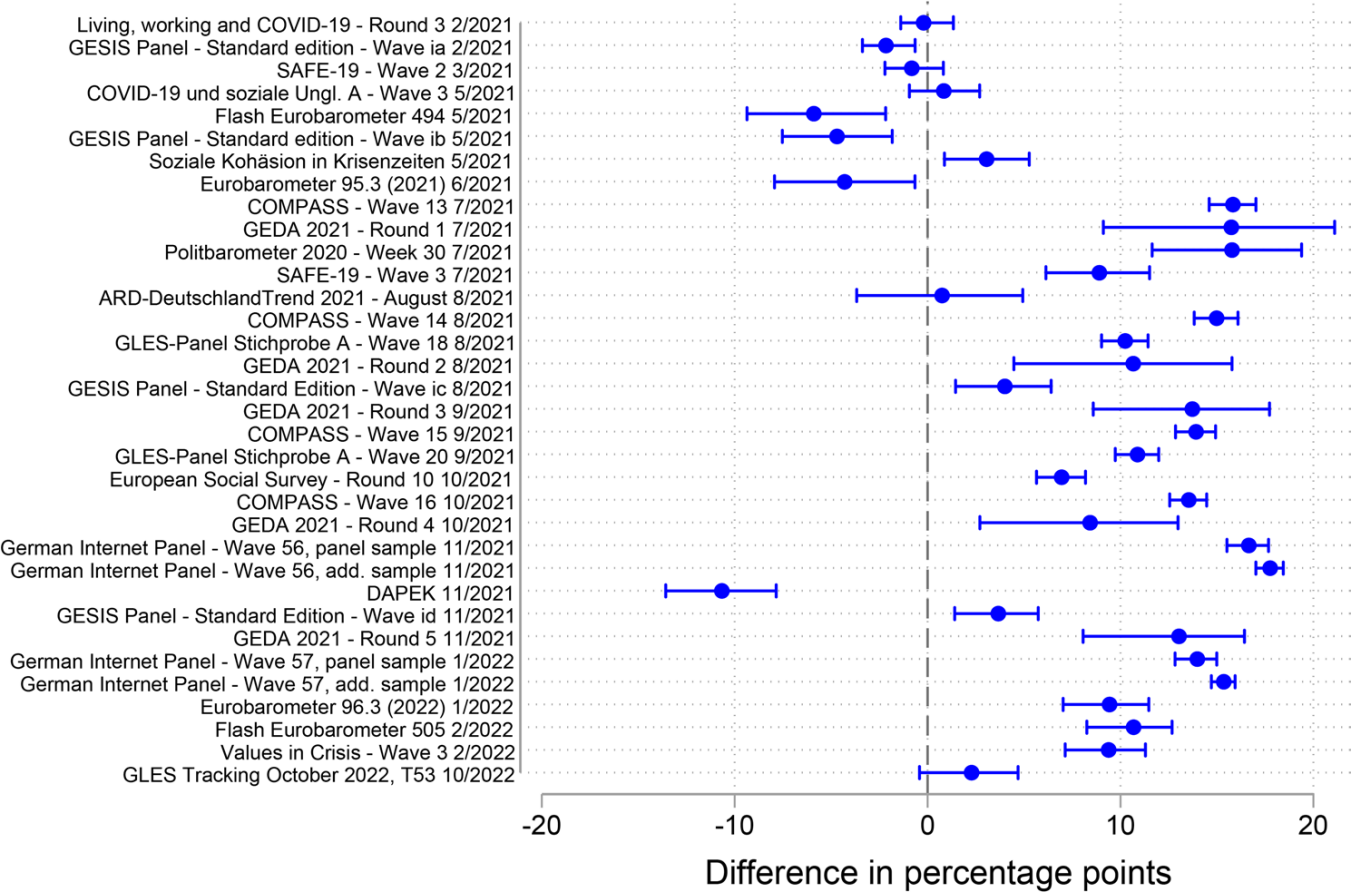
Absolute accuracy of the survey estimates of the COVID-19 vaccination rate with the mean benchmark value over the fieldwork period, age group 18–59 years

Figure 2 shows the absolute accuracy of the survey estimates of the COVID-19 vaccination rate for the age group 18–59 years compared with the mean benchmark value over the fieldwork period. The accuracy varied largely across surveys, ranging from − 10.7% points (the largest underestimation) to + 17.8% points (the largest overestimation). For 29 of the 34 surveys (85%), the confidence intervals did not overlap with zero. This indicates that most surveys exhibited a statistically significant deviation from the benchmark. Specifically, 24 of the 29 surveys that exhibited a statistically significant deviation from the benchmark (83%) overestimated the vaccination rate, and five (17%) underestimated it. The median absolute inaccuracy among all surveys was 9.8% points.

The distribution further revealed that two groups could be distinguished: early surveys (*n* = 8), whose fieldwork began between February and June 2021, and later surveys (*n* = 26), whose fieldwork began in July 2021 or later. Whereas the vaccination rate estimates in the early surveys were relatively more accurate (absolute median = 2.6% points), and seven of the eight surveys in that group underestimated the vaccination rate, the estimates in the later surveys were less accurate (absolute median = 10.8% points), and 23 of the 26 surveys in that group (88%) overestimated the vaccination rate.

In addition to the main analysis, I conducted two robustness checks to validate the results on the accuracy of survey estimates in the age group 18–59 years. For robustness checks RC1 (Fig. S1 in the supplementary material) and RC2 (Fig. S2 in the supplementary material), the comparison of the absolute median accuracy confirmed the conclusion that early surveys were, on average, more accurate than later surveys.

**Fig. 3:**
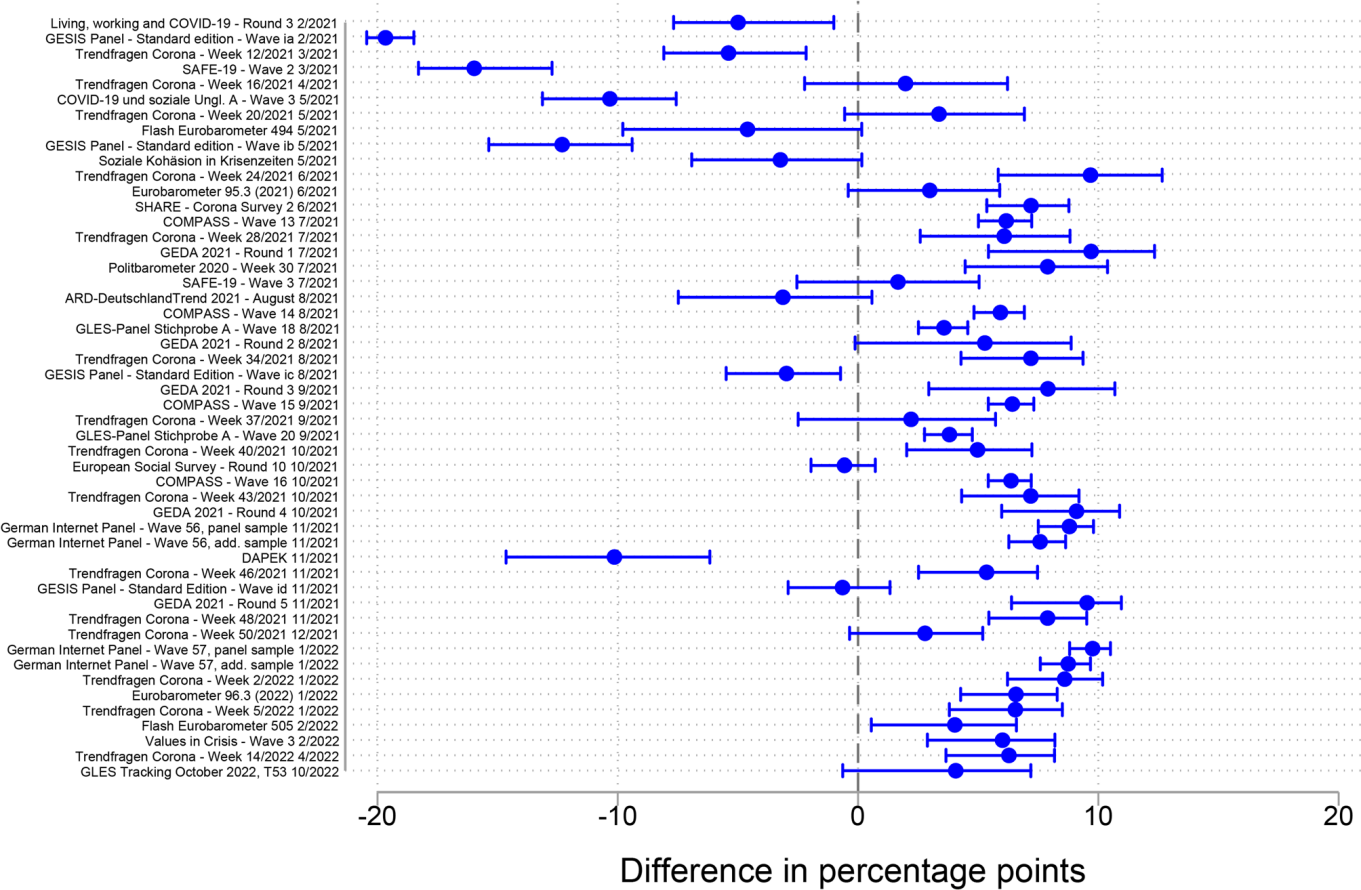
Absolute accuracy of the survey estimates of the COVID-19 vaccination rate compared with the mean benchmark value over the fieldwork period, age group 60 years and older

Figure 3 shows the absolute accuracy of the survey estimates for the age group 60 years and older with the mean benchmark value over the fieldwork period. The magnitude of the range of inaccuracy (− 19.7 to + 9.8% points) was similar to that in the age group 18–59 years, but with greater negative deviations and smaller positive deviations. The median absolute inaccuracy was 6.2% points. For 37 of the 50 surveys included in the analysis (74%), the confidence intervals did not overlap with zero. This indicates that – similarly to the age group 18–59 years – most surveys exhibited a statistically significant deviation from the benchmark. Specifically, of the 37 surveys that exhibited a statistically significant deviation from the benchmark, 29 (78%) overestimated the vaccination rate and eight underestimated it.

Here, too, two groups could be distinguished: early surveys (*n* = 10), whose fieldwork began between February and May 2021, and later surveys (*n* = 40), whose fieldwork began in June 2021 or later. The pattern for the difference between early and later surveys observed in the age group 18–59 years was repeated in the older age group. While all 10 early surveys underestimated the vaccination rate, 29 of the 40 later surveys (73%) overestimated it. In terms of absolute accuracy, early surveys performed slightly better than later surveys (absolute median = 5.2 and 6.3% points, respectively).

To validate these results, I conducted the same robustness checks as in the age group 18–59 years. RC1 (Fig. S3 in the supplementary material) confirmed that the vaccination rate estimates in the early surveys were, on average, more accurate than in later surveys. However, RC2 showed the opposite pattern, with later surveys being more accurate than early surveys. Figure S4 in the supplementary material illustrates that the relative accuracy of the vaccination rate estimates deteriorated especially for the earliest surveys conducted in February and March 2021, a period with comparatively low levels of vaccination.

Overall, I conclude that surveys conducted during the first months of the vaccination campaign mostly underestimated the vaccination rate, whereas surveys conducted during the later phase tended to overestimate it. Furthermore, most of the accuracy measures applied showed that early surveys were more accurate than later ones. However, one of the robustness checks in the age group 60 years and older suggested that the results may depend on the measure applied, as they differed between the standard and relative accuracy measures during months with low vaccination rate levels.

### RQ2 – Association between estimate accuracy and survey design

**Fig. 4:**
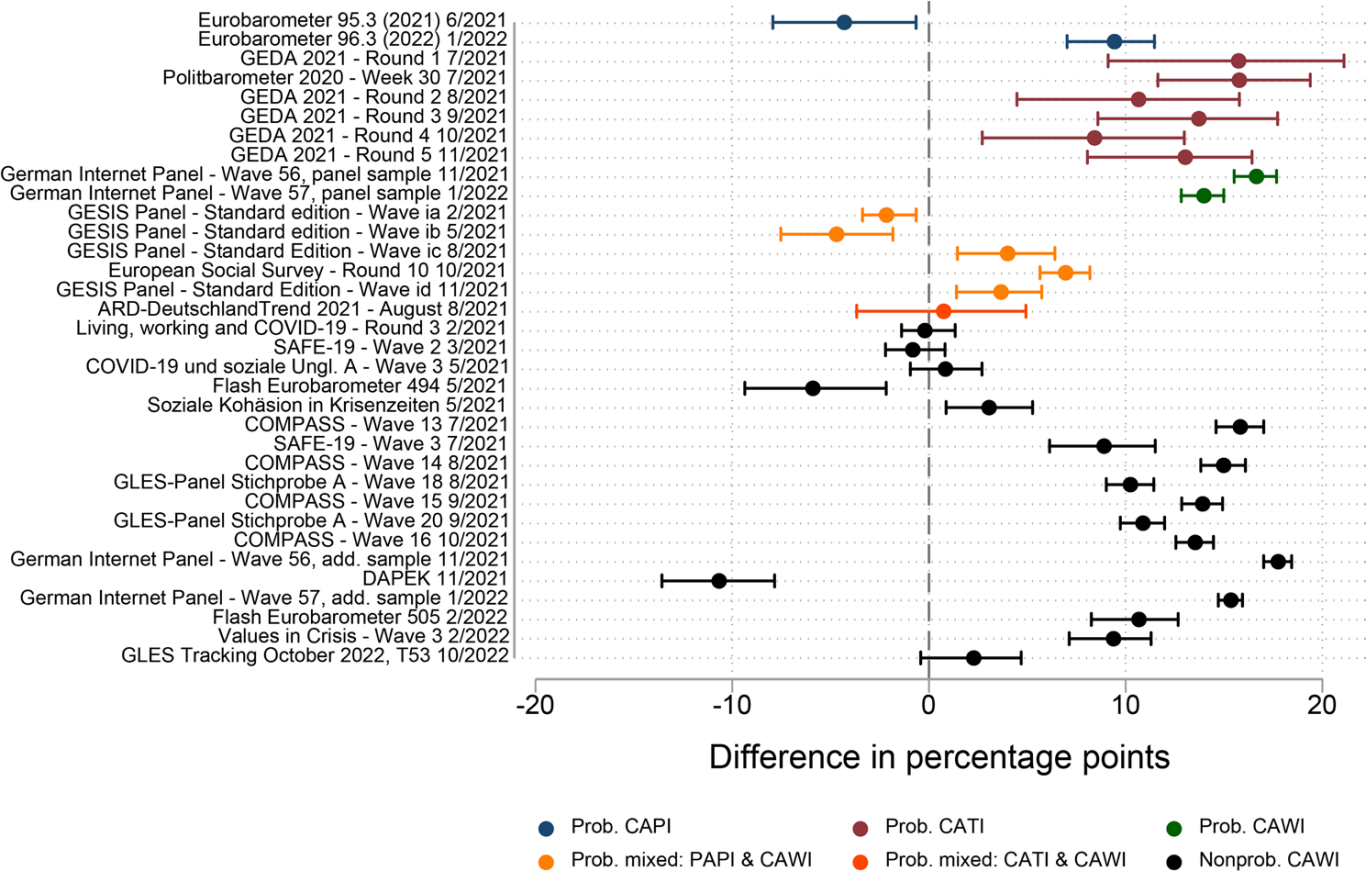
Absolute accuracy of estimates of the COVID-19 vaccination rate by survey design group compared with the mean benchmark value over the fieldwork period, age group 18–59 years

Figure 4 depicts the accuracy of the estimates of the COVID-19 vaccination rate by survey design in the age group 18–59 years. Probability mixed-mode surveys (PAPI & CAWI; CATI & CAWI) achieved the highest absolute accuracy (absolute mean and absolute median = 4% points), followed by the small group of two probability CAPI surveys (absolute mean and absolute median = 7% points). Whereas these two groups and the nonprobability CAWI surveys group displayed both under- and overestimation, all probability CAWI and probability CATI surveys overestimated the vaccination rate. In terms of variability, nonprobability CAWI surveys demonstrated the highest standard deviation (5.7% points). The robustness checks provided similar results in terms of accuracy (see Fig. S5 and Fig. S6 in the supplementary material). Overall, I therefore conclude that, of all survey designs considered in the age group 18–59 years, probability mixed-mode (PAPI & CAWI; CATI & CAWI) surveys and probability CAPI surveys achieved the highest accuracy.

**Fig. 5:**
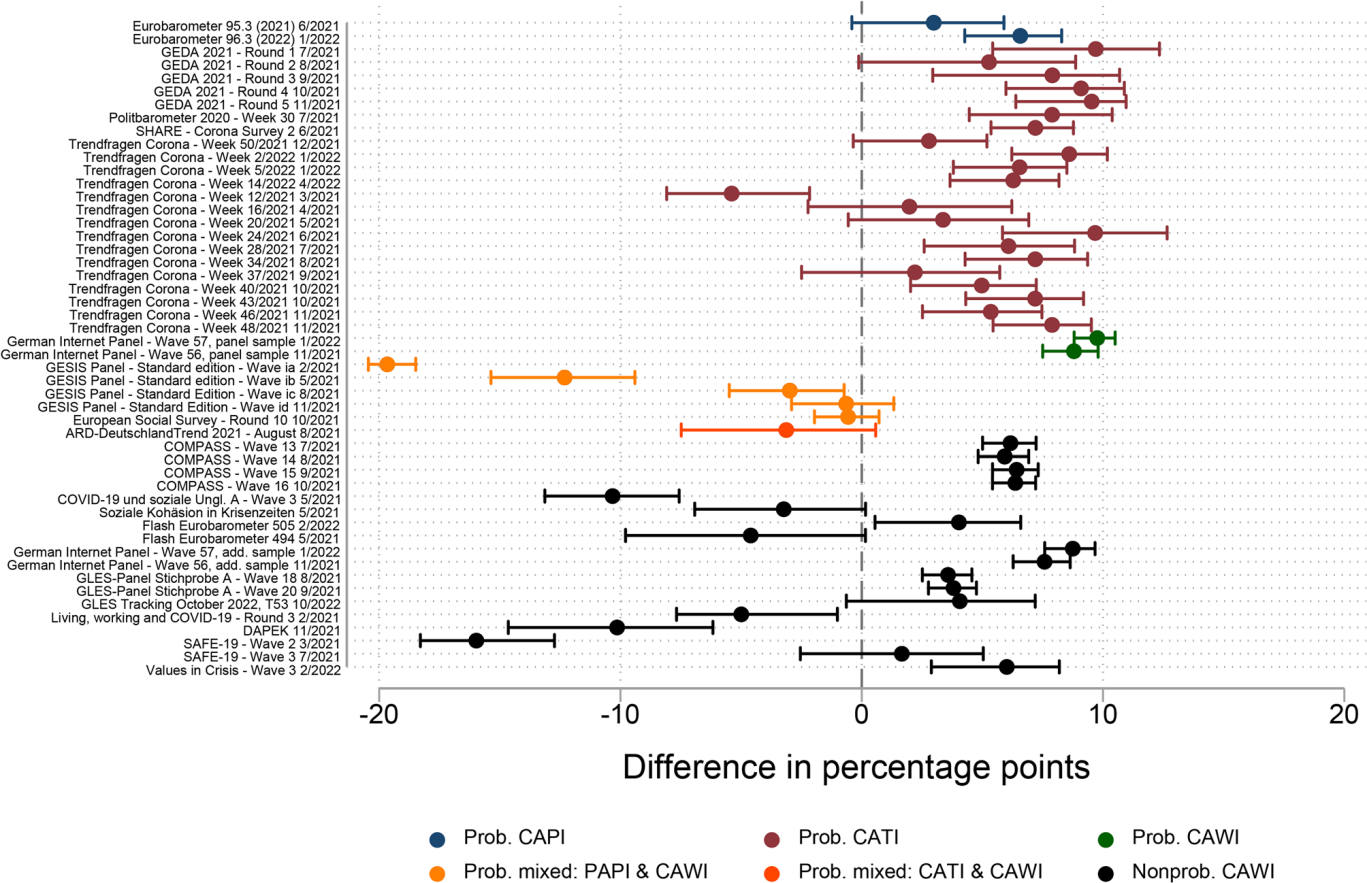
Absolute accuracy of the estimates of the COVID-19 vaccination rate by survey design group compared with the mean benchmark value over the fieldwork period, age group 60 years and older

Figure 5 depicts the absolute accuracy of estimates of the COVID-19 vaccination rate by survey design in the age group 60 years and older. Altogether, the differences in accuracy (measured by absolute mean and absolute median) across survey design groups were comparatively small, with a 4.5%-point gap between the highest and lowest absolute means and a 6.2%-point gap between the highest and lowest absolute medians. As in the case of the age group 18–59 years, the most accurate survey design group in terms of mean accuracy was the probability CAPI surveys group (only two observations, absolute mean and absolute median = 5% points) and the most accurate survey design group in terms of median accuracy was the probability mixed-mode (PAPI & CAWI; CATI & CAWI) surveys group (absolute mean = 7% points, absolute median = 3% points). However, probability mixed-mode surveys were also the survey design group with the highest standard deviation (8% points) and included two cases with some of the highest inaccuracies across all surveys. A possible reason for the relatively large underestimation of the vaccination rate in these two cases may be that the fieldwork took place during the early phase of the vaccination campaign. Among the other survey design groups, overestimation prevailed across probability surveys. Similarly to the age group 18–59 years, nonprobability CAWI surveys displayed high variability, including some of the most accurate survey estimates, as well as comparatively large under- and overestimates. The robustness checks confirmed these findings (Fig. S7 and Fig. S8 in the supplementary material). Overall, the conclusion from the analysis of the age group 18–59 years that probability CAPI surveys and probability mixed-mode (PAPI & CAWI; CATI & CAWI) surveys were the most accurate design groups holds also for the age group 60 years and older, although the differences across survey design groups were relatively small.

### RQ3 – Association between estimate accuracy and adjustment weights

**Fig. 6:**
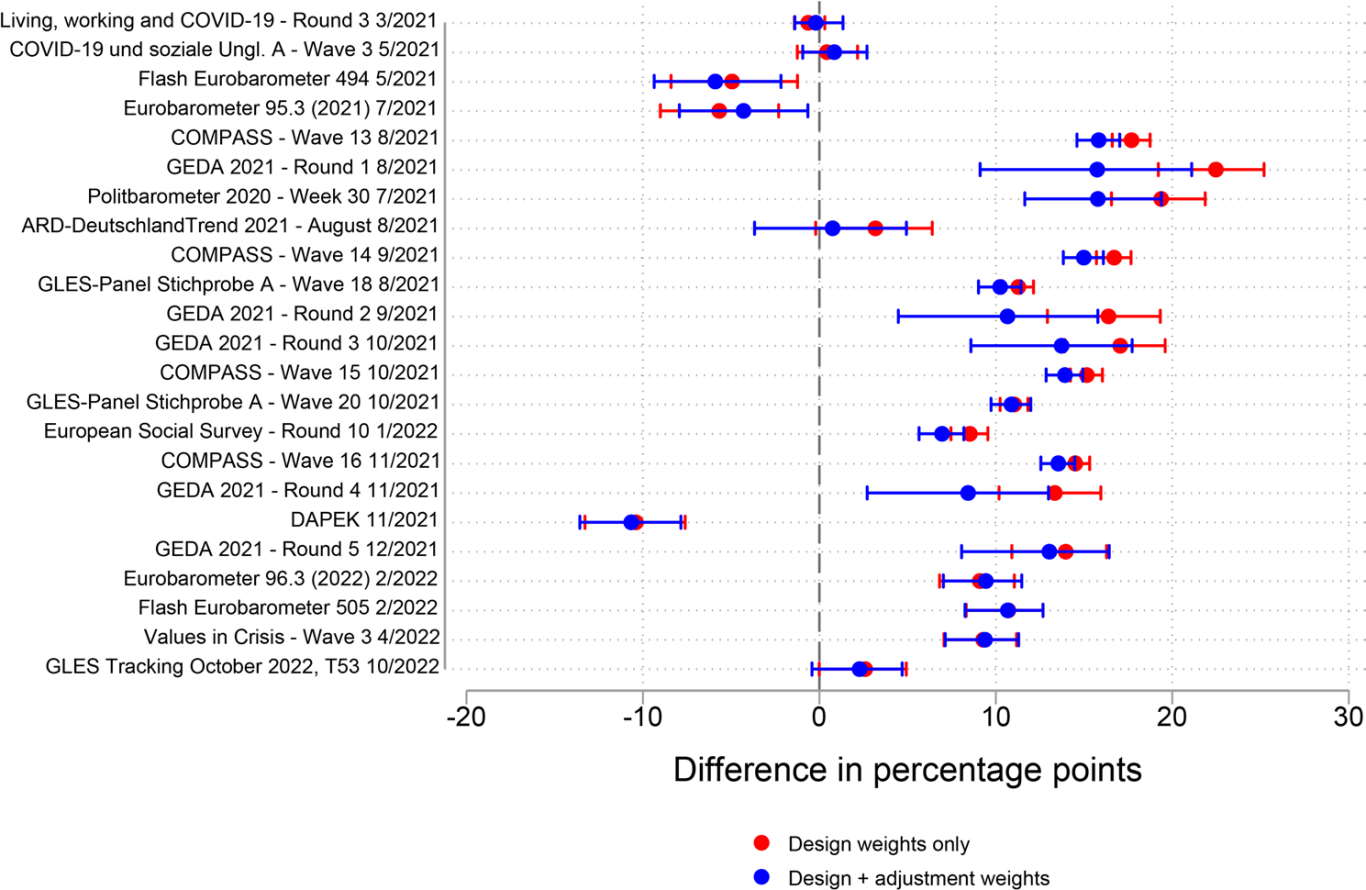
Absolute accuracy of survey estimates of the COVID-19 vaccination rate with and without adjustment weighting compared with the mean benchmark value over the fieldwork period, age group 18–59 years

For the evaluation of the role of adjustment weights for estimate accuracy, I compared survey estimates with only design weights applied (when available) against survey estimates with both design weights (when available) and adjustment weights applied. Figure 6 shows the estimates for absolute accuracy compared with the mean benchmark value over the fieldwork period. The differences in estimates with and without adjustment weighting indicate that for 18 of the 23 surveys, adjustment weights improved accuracy. Measured by the mean absolute accuracy, the estimates with adjustment weights were 2% points more accurate than those without (absolute means = 9 and 11% points, respectively). The robustness checks reached the same conclusion regarding the positive impact of adjustment weights on accuracy (Fig. S9 and Fig. S10 in the supplementary material).

**Fig. 7:**
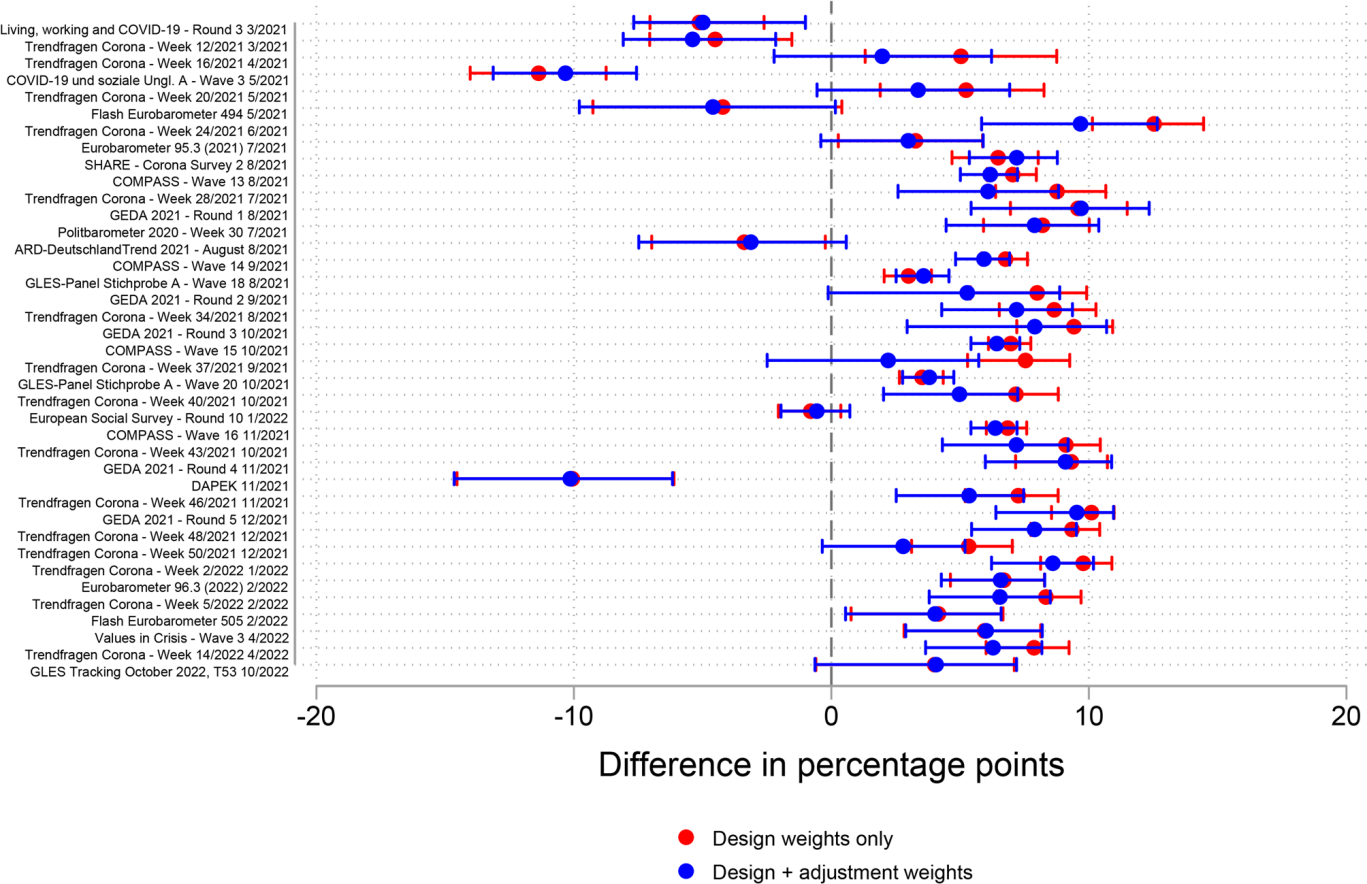
Absolute accuracy of survey estimates of the COVID-19 vaccination rate with and without adjustment weighting compared with the mean benchmark value over the fieldwork period, age group 60 years and older

Figure 7 presents the difference in estimates with and without adjustment weighting for the age group 60 years and older. Similarly to the age group 18–59 years, estimates with adjustment weights were more accurate in 30 of the 39 surveys included in the analysis. The mean difference in absolute accuracy of 1% point in the age group 60 years and older was smaller than that in the younger age group (absolute means = 6 and 7% points, respectively). Robustness checks confirmed the positive impact of adjustment weights on survey accuracy (Fig. S11 and Fig. S12 in the supplementary material).

## Discussion

Surveys are an important source of population health data. Compared with administrative data, they have the advantage of providing comprehensive information on the individual level. However, accuracy is a prerequisite for the reliability of results derived from surveys. To advance research on the data quality of health-related survey measures, the study evaluated the accuracy of survey estimates of the COVID-19 vaccination rate in Germany. For this purpose, I compared the survey estimates with administrative benchmark data (RQ1) and examined whether estimate accuracy differed by survey design (RQ2) and was improved by adjustment weighting (RQ3).

For RQ1, the results showed that the accuracy of survey estimates varied systematically over time. Whereas most surveys conducted in the first months of the vaccination campaign underestimated the vaccination rate, later surveys mostly overestimated it. This pattern indicates a shift in the dominant sources of error over the course of the campaign. The transition from under- to overestimation corresponds directly with the vaccine unrestricted availability for the general population in each age group, with cut-off months of June 2021 for those aged 18–59 years and April 2021 for those aged 60 years and older (see Fig. 1).

The underestimation of the vaccination rate in the early surveys may be explained by the prevalence of representation error, specifically nonresponse bias. During this initial phase, vaccine eligibility was restricted to priority groups, such as the elderly, persons with underlying health conditions, and those in long-term care facilities. These groups were likely more difficult to reach with general population surveys. Consequently, early surveys disproportionately represented the large segment of the population that was not yet eligible and therefore unvaccinated, leading to an overall underestimation of the vaccination rate when compared to the administrative data.

The overestimation that I observed in most surveys in my sample (and especially in those conducted in the later months of the vaccination campaign) aligns with the findings of similar studies on the vaccine uptake in other countries or regions, for example, the United States [46], Sub-Saharan Africa [47], and India [48]. This systematic overestimation can be attributed to multiple types of error.

First, measurement error, particularly social desirability bias, likely played a significant role. As vaccination became a widespread social norm, respondents who were not vaccinated may have untruthfully reported that they were, in order to align with a perceived social expectation. This hypothesis is supported by the results of a survey experiment conducted in Germany during the vaccination campaign [22].

Second, representation errors persisted and contributed to the overestimation. Nonresponse bias due to self-selection was likely present as individuals with a positive attitude toward vaccination may have been more willing to participate in the surveys than those who were vaccine hesitant. Unfortunately, the full extent of this bias typically cannot be measured based on the survey data. Additionally, the language barrier may have been another source of nonresponse bias, as persons without knowledge of German were excluded from surveys that did not offer additional language options. If survey participation correlated with the vaccine uptake in that group, bias may have arisen. This issue was pointed out by the Robert Koch Institute, which analyzed the difference between the vaccination rate estimated based on their administrative data and on a survey they conducted [49].

For RQ2, the analysis revealed differences in accuracy by survey design group – a variable defined based on a combination of sampling procedure and survey mode. Among the survey design groups in my sample, the probability mixed-mode (PAPI & CAWI; CATI & CAWI) surveys and the probability CAPI surveys achieved the highest average accuracy. Although some of the nonprobability surveys also provided comparatively accurate estimates, others did not perform well, resulting in an overall high variance in this group. Rohr et al. [15] found a similar pattern among nonprobability surveys. Overall, my findings based on a large-scale evaluation of individual survey design groups complement the literature on the data quality of health-related surveys, which has so far focused mostly on comparisons of a limited number of survey modes [50–52] or between probability and nonprobability sampling procedures [53–55]. However, further research on the differences between survey design groups is needed, as my analyses do not allow for the identification of determinants of accuracy at the level of the component variables – that is, survey mode and sampling procedure.

Investigating RQ3, I found a positive impact of adjustment weighting on the accuracy of the survey estimates of the COVID-19 vaccination rate. In other words, the sociodemographic variables used to create the adjustment weights in the respective surveys correlated sufficiently with both survey participation and vaccination status. This result is in line with findings from other studies that have demonstrated that weighting enhances the accuracy of various sociodemographic and health-related estimates [25–28]. Given the wide use of adjustment weights in analyses of health survey data [56], I consider further research on the effectiveness of adjustment weighting for specific outcome variables to be necessary.

The study is not without limitations. First, I relied on administrative health data as the best available benchmark, which itself may be prone to errors. In particular, the quality of administrative data on COVID-19 vaccination rates depends on accurate and complete reporting by the institutions responsible for vaccine administration. During the vaccination campaign, the Robert Koch Institute, the publisher of the administrative data on the COVID-19 vaccination rate in Germany, stated that the vaccination rate based on administrative data might be an underestimation of the real rate [49, 57]. Possible reasons for the underestimation included incomplete reporting of one vaccination type by contract physicians (leading to undercounts in certain age groups), limited reporting by occupational physicians, and incomplete transmission of vaccination data from medical practices to the central registry. Although my analyses were based on the most recent and best available version of the administrative data, including corrections made by the Robert Koch Institute, I emphasize the need for caution when interpreting my findings.

Second, the two data types — administrative and survey data — may not fully cover identical populations, which could partly account for differences in vaccination rate estimates. To mitigate this risk, I restricted the analyses to surveys targeting the general resident population in Germany, where vaccination was available to all individuals living, working, or insured in the country regardless of citizenship, and excluded surveys with narrower target groups. Nonetheless, some minor coverage dissimilarities may remain.

Third, the utilized dataset with coded information on surveys does not contain metadata on either the position of the COVID-19 vaccination question within each questionnaire or the overarching survey topic. However, prior research demonstrates that both the placement of survey questions and topic salience can influence data quality through errors related to representation [58–60]. Future research could therefore extend the analysis by examining the role of survey topic (general or COVID-19 specific) and question placement on the accuracy of the survey estimates.

## Conclusions

In this study, I evaluated the accuracy of survey estimates of the COVID-19 vaccination rate in Germany through a comparison with administrative benchmark health data. Based on the results, I conclude that population estimates of the vaccination rate derived from surveys should be interpreted with caution, as inaccuracy — and particularly overestimation — was widely prevalent in the surveys examined. Furthermore, I emphasize the importance of survey design for accuracy. Specifically, I have shown that probability mixed-mode (PAPI & CAWI; CATI & CAWI) and CAPI surveys provided more accurate estimates, on average, than did other designs. Finally, I encourage the use of adjustment weights, as the findings indicate that they have the potential to improve accuracy, as demonstrated by the substantial improvement in most vaccination rate estimates in my study.

## Supplementary Information

Supplementary material 1.

## Data Availability

The analysis in this study is based on multiple data sources. Information on survey design was obtained from the SDCCP 1 dataset, which is publicly available through the GESIS data archive [[https://search.gesis.org/research\_data/SDN-10.7802-2652](https:/search.gesis.org/research_data/SDN-10.7802-2652)]. Individual-level survey data were obtained either from public archives or directly from the authors upon request.Official health statistics on COVID-19 vaccination rates were compiled from two sources: daily vaccination data from the Robert Koch Institute [[https://doi.org/10.5281/zenodo.12697471](https:/doi.org/10.5281/zenodo.12697471)] and population figures from the German Federal Statistical Office [[https://www-genesis.destatis.de/genesis//online? operation=table&code=12411-0009&bypass=true&levelindex=0&levelid=1706611859256#abreadcrumb](https:/www-genesis.destatis.de/genesis/online?operation=table&code=12411-0009&bypass=true&levelindex=0&levelid=1706611859256)].Upon acceptance of the manuscript, replication code will be made available via the GESIS Data Archive for the Social Sciences.

## Abbreviations

CAPI: Computer-assisted personal interviewing
CATI: Computer-assisted telephone interviewing
CAWI: Computer-assisted web interviewing
COVID-19: Coronavirus disease 2019
PAPI: Paper-and-pencil interviewing
RC: Robustness check
RKI: Robert Koch Institute
RQ: Research question

## References

[1] US Census Bureau. Household Pulse Survey: measuring emergent social and economic matters facing U.S. households. United States Census Bureau. 2024. https://www.census.gov/programs-surveys/household-pulse-survey.html. Accessed 23 May 2025.

[2] Australian Bureau of Statistics. Household Impacts of COVID-19 Survey. 2022. https://www.abs.gov.au/statistics/people/people-and-communities/household-impacts-covid-19-survey. 23 May 2025.

[3] Office for National Statistics. Coronavirus (COVID-19) Infection Survey. 2023. https://www.ons.gov.uk/peoplepopulationandcommunity/healthandsocialcare/conditionsanddiseases/bulletins/coronaviruscovid19infectionsurveypilot/previousReleases. Accessed 23 May 2025.

[4] Betsch C, Wieler L, Bosnjak M, Ramharter M, Stollorz V, Omer S, Korn L, Sprengholz P, Felgendreff L, Eitze S, Schmid P, Germany. COVID-19 Snapshot MOnitoring (COSMO Germany): monitoring knowledge, risk perceptions, preventive behaviours, and public trust in the current coronavirus outbreak in Germany [dataset]. PsychArchives. 2020. 10.23668/psycharchives.2776.

[5] Dutwin D, Buskirk TD. 2017. Apples to oranges or Gala versus Golden Delicious? Public Opin Q. 2017;81:213–39. 10.1093/poq/nfw061.

[6] MacInnis B, Krosnick JA, Ho AS, Cho M-J. The accuracy of measurements with probability and nonprobability survey samples: replication and extension. Public Opin Q. 2018;82:707–44. 10.1093/poq/nfy038.

[7] Yeager DS, Krosnick JA, Chang L, Javitz HS, Levendusky MS, Simpser A, et al. Comparing the accuracy of RDD telephone surveys and internet surveys conducted with probability and non-probability samples. Public Opin Q. 2011;75:709–47. 10.1093/poq/nfr020.

[8] Malhotra N, Krosnick JA. The effect of survey mode and sampling on inferences about political attitudes and behavior: comparing the 2000 and 2004 ANES to internet surveys with nonprobability samples. Polit Anal. 2007;15:286–323. 10.1093/pan/mpm003.

[9] Sohlberg J, Gilljam M, Martinsson J. Determinants of polling accuracy: the effect of opt-in internet surveys. Journal of Elections, Public Opinion and Parties. 2017;27:433–47.

[10] Sturgis P, Kuha J, Baker N, Callegaro M, Fisher S, Green J, et al. An assessment of the causes of the errors in the 2015 UK general election opinion polls. J R Stat Soc: Ser Stat Soc. 2018;181:757–81.

[11] Groves RM, Fowler FJ Jr, Couper MP, Lepkowski JM, Singer E, Tourangeau R. Survey methodology. 2nd ed. Hoboken, NJ: Wiley; 2009.

[12] Groves RM, Lyberg L. Total survey error: past, present, and future. Public Opin Q. 2010;74:849–79.

[13] Lyberg LE, Weisberg HF. Total survey error: a paradigm for survey methodology. In: Wolf C, Joy D, Smith TW, Fu Y-c, editors. The SAGE handbook of survey methodology. 2016. London: Sage Publications Ltd.; 2016. pp. 27–40.

[14] Chang L, Krosnick JA. National surveys via RDD telephone interviewing versus the internet: comparing sample representativeness and response quality. Public Opin Q. 2009;73:641–78.

[15] Rohr B, Silber H, Felderer B. Comparing the accuracy of univariate, bivariate, and multivariate estimates across probability and nonprobability surveys with population benchmarks. Sociol Methodol. 2024. 10.1177/00811750241280963.

[16] Cornesse C, Bosnjak M. Is there an association between survey characteristics and representativeness? A meta-analysis. Surv Res Methods. 2018. 10.18148/srm/2018.v12i1.7205.

[17] Blom AG, Herzing JME, Cornesse C, Sakshaug JW, Krieger U, Bossert D. Does the recruitment of offline households increase the sample representativeness of probability-based online panels? Evidence from the German internet panel. Soc Sci Comput Rev. 2017;35:498–520.

[18] Leenheer J, Scherpenzeel A. Does it pay off to include non-Internet households in an internet panel? Int J Internet Sci. 2013;8:17–29.

[19] Revilla M, Cornilleau A, Cousteaux A-S, Legleye S, de Pedraza P. What is the gain in a probability-based online panel of providing internet access to sampling units who previously had no access? Soc Sci Comput Rev. 2016;34(4):479–96.

[20] Henderson C, Evans-Lacko S, Flach C, Thornicroft G. Responses to mental health stigma questions: the importance of social desirability and data collection method. Can J Psychiatry. 2012;57:152–60. 22398001 10.1177/070674371205700304

[21] Reisinger J. Subjective well-being and social desirability. J Public Econ. 2022. 10.1016/j.jpubeco.2022.104745.

[22] Wolter F, Mayerl J, Andersen HK, Wieland T, Junkermann J. Overestimation of COVID-19 vaccination coverage in population surveys due to social desirability bias: results of an experimental methods study in Germany. Socius Sociol Res Dyn World. 2022. 10.1177/23780231221094749.

[23] Felderer B, Kirchner A, Kreuter F. The effect of survey mode on data quality: disentangling nonresponse and measurement error bias. J Off Stat. 2019;35:93–115.

[24] Sakshaug JW, Beste J, Trappmann M. Effects of mixing modes on nonresponse and measurement error in an economic panel survey. J Labour Market Res. 2023. 10.1186/s12651-022-00328-1.

[25] Berrens RP, Bohara AK, Jenkins-Smith H, Silva C, Weimer DL. The advent of internet surveys for political research: a comparison of telephone and internet samples. Polit Anal. 2003. 10.1093/pan/11.1.1.

[26] Jensen HAR, Lau CJ, Davidsen M, Feveile HB, Christensen AI, Ekholm O. The impact of non-response weighting in health surveys for estimates on primary health care utilization. Eur J Public Health. 2022;32:450–5. 35373254 10.1093/eurpub/ckac032 PMC9159316

[27] Steinmetz S, Bianchi A, Tijdens K, Biffignandi S. Improving web survey quality. In: Callegaro M, Baker R, Bethlehem J, Göritz AS, Krosnick JA, Lavrakas PJ, editors. Online panel research. London: John Wiley & Sons, Ltd; 2014. pp. 273–98.

[28] Wang W, Rothschild D, Goel S, Gelman A. Forecasting elections with non-representative polls. Int J Forecast. 2015;31:980–91.

[29] Copas A, Burkill S, Conrad F, Couper MP, Erens B. An evaluation of whether propensity score adjustment can remove the self-selection bias inherent to web panel surveys addressing sensitive health behaviours. BMC Med Res Methodol. 2020. 10.1186/s12874-020-01134-4. 33032535 10.1186/s12874-020-01134-4 PMC7545552

[30] Haddad C, Sacre H, Zeenny RM, Hajj A, Akel M, Iskandar K, et al. Should samples be weighted to decrease selection bias in online surveys during the COVID-19 pandemic? Data from seven datasets. BMC Med Res Methodol. 2022. 10.1186/s12874-022-01547-3. 35249541 10.1186/s12874-022-01547-3 PMC8898325

[31] Hlatshwako TG, Shah SJ, Kosana P, Adebayo E, Hendriks J, Larsson EC, et al. Online health survey research during COVID-19. Lancet Digit Health. 2021. 10.1016/S2589-7500(21)00002-9. 33509387 10.1016/S2589-7500(21)00002-9 PMC10000261

[32] Lin Y-H, Chen C-Y, Wu S-I. Efficiency and quality of data collection among public mental health surveys conducted during the COVID-19 pandemic: systematic review. J Med Internet Res. 2021. 10.2196/25118. 33481754 10.2196/25118 PMC7879724

[33] Pierce M, McManus S, Jessop C, John A, Hotopf M, Ford T, et al. Says who? The significance of sampling in mental health surveys during COVID-19. Lancet Psychiatry. 2020;7:567–8. 10.1016/S2215-0366(20)30237-6. 32502467 10.1016/S2215-0366(20)30237-6 PMC7266586

[34] von Glasenapp K, Skora T, Gummer T, Naumann E. Survey design and quality during the COVID-19 pandemic in Germany: An assessment with 686 social science surveys. Survey Research Methods 2025 forthcoming. 10.1038/s41597-024-03475-x PMC11169371 38866799

[35] Federal Ministry of Health. Verordnung zum Anspruch auf Schutzimpfung gegen das Coronavirus SARS-CoV-2 (Coronavirus-Impfverordnung-CoronaImpfV) vom 18. Dezember 2020. 2020. https://www.bundesanzeiger.de/pub/publication/uiOU7Q0UIHTjQ7Uk9S2/content/uiOU7Q0UIHTjQ7Uk9S2/BAnz%20AT%2021.12.2020%20V3.pdf?inline. Accessed 15 August 2025.

[36] Federal Ministry of Health. Chronik zum Coronavirus SARS-CoV-2. 2023. https://www.bundesgesundheitsministerium.de/coronavirus/chronik-coronavirus.html?stand=20210104&cHash=c2dc31e1a7befe544dae855bbcc57509. Accessed 23 May 2025.

[37] Federal Ministry of Health. Verordnung zum Anspruch auf Schutzimpfung gegen das Coronavirus SARS-CoV-2 (Coronavirus-Impfverordnung-CoronaImpfV) vom 1. Juni 2021. 2021. https://www.bundesanzeiger.de/pub/publication/eAOaquujTaFsA5RvNYF/content/eAOaquujTaFsA5RvNYF/BAnz%20AT%2002.06.2021%20V2.pdf?inline

[38] Robert Koch Institute. COVID-19-Impfungen in Deutschland (Version 2024-07-09T09:00:15 + 02:00) [Data set]. 2024. 10.5281/zenodo.12697471

[39] Federal Statistical Office (Destatis). Tables 12411-0009: Bevölkerung: Deutschland, Stichtag, Geschlecht, Altersgruppen, Staatsangehörigkeit [Dataset]. 2024 https://www-genesis.destatis.de/genesis//online?operation=table&code=12411-0009&bypass=true&levelindex=0&levelid=1706611859256#abreadcrumb. Accessed 23 May 2025.

[40] Hale T, Angrist N, Goldszmidt R, Kira B, Petherick A, Phillips T, et al. A global panel database of pandemic policies (Oxford COVID-19 government response tracker). Nat Hum Behav. 2021;5:529–38. 33686204 10.1038/s41562-021-01079-8

[41] Robert Koch Institute. Epidemiologisches Bulletin 49/2021. 2021c. https://www.rki.de/DE/Aktuelles/Publikationen/Epidemiologisches-Bulletin/2021/49_21.pdf?__blob=publicationFile&v=3. Accessed 23 May 2025.

[42] Gummer T, Skora T, von Glasenapp K, Naumann E. A dataset on survey designs and quality of social and behavioral science surveys during the COVID-19 pandemic. Sci Data. 2024. 10.1038/s41597-024-03475-x. 38866799 10.1038/s41597-024-03475-x PMC11169371

[43] von Glasenapp K, Skora T, Gummer T, Naumann E. SDCCP 1—Survey design and data quality during the Covid-19 pandemic. Dataset. 2024. 10.7802/2652. 10.1038/s41597-024-03475-x PMC11169371 38866799

[44] Schwerdtfeger M, Kummerow M, Weyandt K. GESIS Panel wave report: wave ia. GESIS – Leibniz Institute for the Social Sciences. 2022. 10.4232/1.13880

[45] Wolf C, Christmann P, Gummer T, Schnaudt C, Verhoeven S. Conducting general social surveys as self-administered mixed-mode surveys. Public Opin Q. 2021;85:623–48.

[46] Bradley VC, Kuriwaki S, Isakov M, Sejdinovic D, Meng X-L, Flaxman S. Unrepresentative big surveys significantly overestimated US vaccine uptake. Nature. 2021. 10.1038/s41586-021-04198-4. 34880504 10.1038/s41586-021-04198-4 PMC8653636

[47] Markhof Y, Wollburg P, Zezza A. Are vaccination campaigns misinformed? Experimental evidence from COVID-19 in low- and middle-income countries. World Bank. 2023. 10.1596/1813-9450-10443.

[48] Yang Y, Dempsey W, Han P, Deshmukh Y, Richardson S, Tom B, et al. Exploring the big data paradox for various estimands using vaccination data from the global COVID-19 trends and impact survey (CTIS). Sci Adv. 2024. 10.1126/sciadv.adj0266. 38820165 10.1126/sciadv.adj0266 PMC11314312

[49] Robert Koch Institute. COVID 19 Impfquoten-Monitoring in Deutschland (Covimo) Report 7. 2021b. https://www.rki.de/DE/Themen/Infektionskrankheiten/Impfen/Forschungsprojekte/abgeschlossene-Projekte/COVIMO/Downloads/covimo_studie_bericht_7.html. Accessed 23 May 2025.

[50] Christensen AI, Ekholm O, Glümer C, Juel K. Effect of survey mode on response patterns: comparison of face-to-face and self-administered modes in health surveys. Eur J Public Health. 2014;24:327–32. 23766339 10.1093/eurpub/ckt067

[51] Hoebel J, von der Lippe E, Lange C, Ziese T. Mode differences in a mixed-mode health interview survey among adults. Arch Public Health. 2014. 10.1186/2049-3258-72-46. 25810913 10.1186/2049-3258-72-46 PMC4373057

[52] Milton AC, Ellis LA, Davenport TA, Burns JM, Hickie IB. Comparison of self-reported telephone interviewing and web-based survey responses: findings from the second Australian young and well national survey. JMIR Ment Health. 2017. 10.2196/mental.8222. 28951382 10.2196/mental.8222 PMC5635234

[53] Bethell C, Fiorillo J, Lansky D, Hendryx M, Knickman J. Online consumer surveys as a methodology for assessing the quality of the United States health care system. J Med Internet Res. 2004. 10.2196/jmir.6.1.e2. 15111268 10.2196/jmir.6.1.e2 PMC1550587

[54] Erens B, Burkill S, Couper MP, Conrad F, Clifton S, Tanton C, et al. Nonprobability web surveys to measure sexual behaviors and attitudes in the general population: a comparison with a probability sample interview survey. J Med Internet Res. 2014. 10.2196/jmir.3382. 25488851 10.2196/jmir.3382 PMC4275497

[55] Legleye S, Charrance G, Razafindratsima N, Bajos N, Bohet A, Moreau C. The use of a nonprobability internet panel to monitor sexual and reproductive health in the general population. Sociol Methods Res. 2018;47:314–48.

[56] Bell BA, Onwuegbuzie AJ, Ferron JM, Jiao QG, Hibbard ST, Kromrey JD. Use of design effects and sample weights in complex health survey data: a review of published articles using data from 3 commonly used adolescent health surveys. Am J Public Health. 2012. 10.2105/AJPH.2011.300398. 22676502 10.2105/AJPH.2011.300398 PMC3477989

[57] Robert Koch-Institut. COVID 19 Impfquoten-Monitoring in Deutschland (Covimo) Report 6. 2021a. https://www.rki.de/DE/Themen/Infektionskrankheiten/Impfen/Forschungsprojekte/abgeschlossene-Projekte/COVIMO/Downloads/covimo_studie_bericht_6.html. Accessed 23 May 2025.

[58] Groves RM, Presser S, Dipko S. The role of topic interest in survey participation decisions. Public Opin Q. 2004;68:2–31. 10.1093/poq/nfh002.

[59] Plutowski L, Zechmeister EJ. (2024). Do Question Topic and Placement Shape Survey Breakoff Rates? Survey Methods: Insights from the Field (SMIF). 2024. 10.13094/SMIF-2024-00005.

[60] Keusch F. The role of topic interest and topic salience in online panel web surveys. Int J Market Res. 2013;55:59–80. 10.2501/IJMR-2013-007.

