# Supplementary material for: Evaluating the accuracy of survey data: a case study of COVID-19 vaccination rates in Germany
Source: BMC Med Res Methodol. 2025 Oct 22;25:234. doi: 10.1186/s12874-025-02702-2 (PMC12541969; doi:10.1186/s12874-025-02702-2)
Supplement: Supplementary file 1 — Supplementary material 1. [file 12874_2025_2702_MOESM1_ESM.docx]

**Supplementary Material**

**Fig. S1** Absolute accuracy of survey estimates of the COVID-19 vaccination rate compared with the mean benchmark value over the fieldwork period (standard measure) and the benchmark value on the first day of fieldwork (robustness check 1), age group 18–59 years.

**Fig. S2** Absolute accuracy of the survey estimates of the COVID-19 vaccination rate in Germany compared with the mean benchmark value over the fieldwork period (standard measure) and relative accuracy compared with the mean benchmark value over the fieldwork period (robustness check 2), age group 18–59 years.

**Fig. S3** Absolute accuracy of the survey estimates of the COVID-19 vaccination rate compared with the mean benchmark value over the fieldwork period (standard measure) and with the benchmark value on the first day of fieldwork (robustness check 1), age group 60 years and older.

**Fig. S4** Absolute accuracy of the survey estimates of the COVID-19 vaccination rate in Germany compared with the mean benchmark value over the fieldwork period (standard measure) and relative accuracy compared with the mean benchmark value over the fieldwork period (robustness check 2), age group 60 years and older.

**Fig. S5** Absolute accuracy of the survey estimates of the COVID-19 vaccination rate in Germany by survey design group compared with the benchmark value on the first day of fieldwork (robustness check 1), age group 18–59 years.

**Fig. S6** Relative accuracy of the survey estimates of the COVID-19 vaccination rate in Germany by survey design group compared with the mean benchmark value over the fieldwork period (robustness check 2), age group 18–59 years.

**Fig. S7** Absolute accuracy of the survey estimates of the COVID-19 vaccination rate in Germany by survey design group compared with the benchmark value on the first day of fieldwork (robustness check 1), age group 60 years and older.

**Fig. S8** Relative accuracy of the survey estimates of the COVID-19 vaccination rate in Germany by survey design group compared with the mean benchmark value over the fieldwork period (robustness check 2), age group 60 years and older.

**Fig. S9** Absolute accuracy of the survey estimates of the COVID-19 vaccination rate in Germany with and without adjustment weighting compared with the benchmark value on the first day of fieldwork (robustness check 1), age group 18–59 years.

**Fig. S10** Relative accuracy of the survey estimates of the COVID-19 vaccination rate in Germany with and without adjustment weighting compared with the mean benchmark value over the fieldwork period (robustness check 2), age group 18–59 years.

**Fig. S11** Absolute accuracy of the survey estimates of the COVID-19 vaccination rate in Germany with and without adjustment weighting compared with the benchmark value on the first day of fieldwork (robustness check 1), age group 60 years and older.

**Fig. S12** Relative accuracy of the survey estimates of the COVID-19 vaccination rate in Germany with and without adjustment weighting compared with the mean benchmark value over the fieldwork period (robustness check 2), age group 60 years and older.
